# Stronger Prejudices Are Associated With Decreased Model-Based Control

**DOI:** 10.3389/fpsyg.2021.767022

**Published:** 2022-01-05

**Authors:** Miriam Sebold, Hao Chen, Aleyna Önal, Sören Kuitunen-Paul, Negin Mojtahedzadeh, Maria Garbusow, Stephan Nebe, Hans-Ulrich Wittchen, Quentin J. M. Huys, Florian Schlagenhauf, Michael A. Rapp, Michael N. Smolka, Andreas Heinz

**Affiliations:** ^1^Department of Psychiatry and Psychotherapy, Charité – Universitätsmedizin Berlin, Corporate member of Freie Universität Berlin, Humboldt-Universität zu Berlin, and Berlin Institute of Health, Berlin, Germany; ^2^Department for Social and Preventive Medicine, University of Potsdam, Potsdam, Germany; ^3^Department of Psychiatry, Neuroimaging Center, Technische Universität Dresden, Dresden, Germany; ^4^Institute of Clinical Psychology and Psychotherapy, Technische Universität Dresden, Dresden, Germany; ^5^Department of Child and Adolescent Psychiatry, Faculty of Medicine, Technische Universität Dresden, Dresden, Germany; ^6^Department of Economics, Zurich Center for Neuroeconomics, University of Zurich, Zurich, Switzerland; ^7^Department of Psychiatry and Psychotherapy, Ludwig-Maximilians-Universität München, Munich, Germany; ^8^Division of Psychiatry, University College London, London, United Kingdom; ^9^Max Planck UCL Centre for Computational Psychiatry and Ageing Research, University College London, London, United Kingdom; ^10^Max Planck Institute for Human Cognitive and Brain Sciences, Leipzig, Germany

**Keywords:** subtle and blatant prejudice, immigrant, social behavior, decision-making, computational modeling, reinforcement learning

## Abstract

**Background:** Prejudices against minorities can be understood as habitually negative evaluations that are kept in spite of evidence to the contrary. Therefore, individuals with strong prejudices might be dominated by habitual or “automatic” reactions at the expense of more controlled reactions. Computational theories suggest individual differences in the balance between habitual/model-free and deliberative/model-based decision-making.

**Methods:** 127 subjects performed the two Step task and completed the blatant and subtle prejudice scale.

**Results:** By using analyses of choices and reaction times in combination with computational modeling, subjects with stronger blatant prejudices showed a shift away from model-based control. There was no association between these decision-making processes and subtle prejudices.

**Conclusion:** These results support the idea that blatant prejudices toward minorities are related to a relative dominance of habitual decision-making. This finding has important implications for developing interventions that target to change prejudices across societies.

## Introduction

“I am a German when we win, but I am an immigrant when we lose,” said Mesut Özil, a FIFA world cup winner, who quit the German soccer team in 2018 due to alleged disrespect and racism. And Özil is not alone. Racism, prejudice and stigma are major problems experienced by migrants worldwide. In Germany 3 million persons of Turkish descent represent the largest minority group. A recent study revealed that in Germany, job candidates of Turkish origin are less likely to receive an invitation to a job interview compared to equally qualified candidates of German origin (Thijssen et al., 2019). Moreover, teachers give poorer grades to students with a Turkish name compared to their German counterparts despite equal performance (Bonefeld and Dickhauser, 2018). Prejudices thus impair social participation and challenge social cohesion.

Per definition, prejudices include “biases” of perception and evaluations toward outgroup members and are a core mechanism underlying discrimination, which significantly impacts the safety and well-being of vulnerable groups worldwide (Mattan et al., 2018). In his seminal work, Allport (1954) defines prejudice as “an antipathy based upon faulty and inflexible generalization.” He emphasizes that categorization – the process underlying prejudice – is an “essential human least effort” to handle sensory and/or cognitive overload. Despite being more than 60 years old, these statements are still relevant: they frame prejudices within a cognitive perspective, and suggest a role for cognitive processes where information is *automatically* processed (Devine, 2001). This framework additionally suggests that explicit cognitive control can prevent the expression of a person’s judgment and behavior toward minorities or other outgroups. Thus, from a cognitive perspective, expressed biases against minorities may at times represent an overactive inflexible “automatic system” at the expense of a more deliberative “control system” (Devine, 1989). During the past decades, a number of studies from social psychology have investigated whether prejudices relate to an overactive automatic control system and/or a disrupted deliberative control system. In fact, there is evidence for both associations.

For instance, racial biases and stereotypes can be primed (Kawakami et al., 1999; Payne, 2001), suggesting that prejudices stem from an automatic control system. Likewise, the implicit association test, one of the most frequently used measures of implicit racial biases, relies on the assumption that prejudices reflect automatic associations (Greenwald et al., 1998).

On the other hand, deliberative control systems can interfere with prejudices. For instance, subjects with high prejudices are impaired in inhibiting stereotype-congruent thoughts and replacing them with thoughts reflecting equality and negations of the stereotype (Devine, 1989). Likewise, the internal motivation to control prejudices is a mechanism to suppress prejudices and individuals who score low on this measures show stronger implicit racial biases (Plant and Devine, 1998). Cognitive load can further increase prejudices in these subjects (Park et al., 2008). Moreover, there is also some evidence that subjects with low cognitive control capacities are at risk to show increased racial biases (Amodio et al., 2004; Payne, 2005). For instance, in a study by Amodio et al. (2004) individuals with weak electrophysiological signals indicative of performance monitoring and cognitive control also showed decreased behavioral adaptation after racially biased responses. Moreover, White subjects with high levels of prejudices displayed pronounced impairments in cognitive control after encountering an interaction with a Black confederate (Richeson and Shelton, 2003), suggesting a negative association between prejudices and deliberate control resources.

However, while these studies indicate that prejudices might be associated with increased automatic responses and/or decreased deliberative control resources, it has been difficult to tease apart the contribution of both control modes. The only tool that, to date, has offered critical leverage to teasing apart these contributions is the process-dissociation procedure (Payne et al., 2005). This procedure involves arranging experiments so that in some conditions automatic and controlled processes lead participants to make the same response, whereas in other conditions they lead to different responses. By placing automatic and controlled processes both in concert and in opposition, one can thus measure the unique contribution of each process (Lambert et al., 2003). One previous study used this procedure and demonstrated that increased expression of prejudices toward minorities in public settings were associated with a decrease in cognitive control rather than increases in automatic heuristics (Lambert et al., 2003).

One additional tool that enables to tease apart the relative contribution of the automatic and the deliberative control systems is computational modeling of task behavior. Computational accounts of decision-making have proposed the existence of two control modes that guide decision-making (Daw et al., 2011). These modes resemble the automatic-controlled dichotomy of the cognitive account: a model-free, presumably habitual control system that is associative in nature, and a model-based, goal-directed control system that prospectively plans actions based on using a model of the environment (Daw et al., 2011; Friedel et al., 2014). The profound advantage of this computational account is, that it is based on theory-driven reinforcement learning (RL) models and therefore provides a mechanistic understanding of the underlying decision-making processes. The task that has been most widely used to investigate the individual contribution of model-free and model-based decision-making is the two-step task (Daw et al., 2011). By using this task, it has been shown that humans show both control modes, albeit with intraindividual variance and interindividual differences regarding the balance between both decision-making components (Doll et al., 2012; Sebold et al., 2016).

Here, we used the two-step task to quantify model-free and model-based control in a rather large cohort of subjects (*n* = 127), and investigated whether these measures were associated with self-reported overt (blatant) or hidden (subtle) prejudices. As outlined above, in Germany one of the most stigmatized minorities are migrants from Turkey (Barwick and Beaman, 2019; Thijssen et al., 2019), and we therefore assessed prejudices against this outgroup by using the German version of the Blatant and Subtle Prejudice Scale [BSPS; Pettigrew and Meertens (1995)].

## Materials and Methods

### Subjects and Procedure

This study was part of a longitudinal prospective study to identify learning and decision-making mechanisms underlying dysfunctional alcohol consumption during early young adulthood, which is not of interest in this report (LEAD-study, ClinicalTrials.gov identifier: NCT01744834). Subjects were randomly sampled from the population in two German cities (Berlin and Dresden) and had no history of neurological or psychiatric diseases. At age 21 (Follow-up time point year 3) we additionally aimed to use this sample of young men to assess the association between empathy, alexithymia and the self-reported level of prejudices (Önal et al., 2021). Therefore, we asked the subjects to complete – among other computerized questionnaires – the Blatant and Subtle Prejudice Scale (Pettigrew and Meertens, 1995). In a separate session (13 ± 14 days) all subjects additionally performed the two- step task by means to assess the relative contribution of model-free vs. model-based control. Subjects were compensated for their participation with a fixed amount for the appointment (10 EUR/hour) plus an additional sum contingent on task performance (max. 10 EUR).

For the testing of our hypotheses (namely, whether model-free/model-based control was associated with blatant and/or subtle prejudices), we included only data of individuals who had provided complete questionnaire and task data. Originally the sample consisted of 132 subjects. However, *n* = 1 participant did not fill out the Blatant and Subtle questionnaire, and the behavioral data of the two-step task of *n* = 4 subjects were excluded based on technical problems. Characteristics of the final sample (total *n* = 127; *n* = 60 from Berlin and *n* = 67 from Dresden) are outlined in Table 1.

**TABLE 1:** Sample description.

	Total sample (*n* = 127, only male)		
	*N*	Mean	SD
***Demographic/Clinical***			
Site (Berlin/Dresden)	127 (60/67)		
Age, years	127	21.50	0.25
School education, years	125	12.2	1.11
Migration background in %*	115	21.7	
Impulsivity**	127	28.8	5.14
***Prejudice***			
Blatant prejudice	127	15.40	5.4
Threat/Reject	127	9.73	3.75
Intimacy	127	5.67	2.13
Subtle prejudice	127	30.65	7.75
Traditional values	127	9.83	3.78
Cultural differences	127	14.16	3.67
Positive emotions	127	6.65	2.28
***Cognitive performance***			
Digit span backward	127	10.77	2.84
Digit symbol substitution test	127	11.45	2.67

*Defined as one self, mother, father and/or one of the grandparents being born in a country other than Germany.

**As assessed by the Barratt Impulsiveness Scale.

### Two-Step Task

**FIGURE 1:**
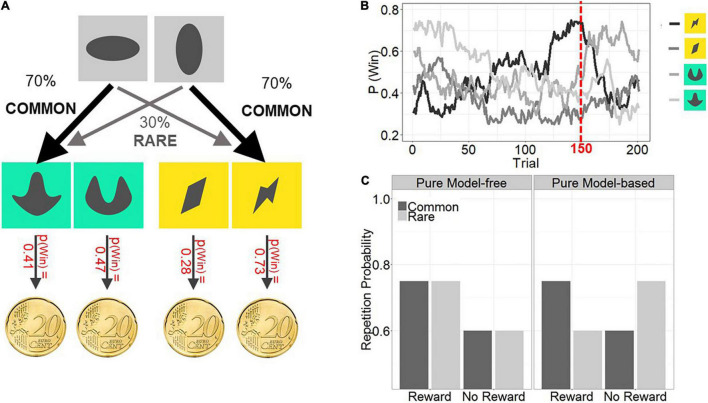
**(A)** Trial configuration: In each trial, subjects had to make two consecutive choices. At a first stage (gray boxes), subjects chose one stimulus over the other (e.g., horizontal ellipse over vertical ellipse) and then proceeded to a second stage (colored boxes: either green or yellow stimulus pair) where they chose between two stimuli. Second stage choices were probabilistically rewarded or punished according to Gaussian random walks. In the depicted example the most right stimulus has a 73% of being rewarded. Transition probabilities between first and second stages varied for first stage choices: Whereas one stimulus choice led commonly (70% of all trials) to one second stage stimulus pair and rarely (30% of all trials) to the second stage stimulus pair, the opposite was true for the other first stage choice. **(B)** Reward probabilities for second stage choices. The example in **(A)** are the outcome probabilities for all four second stage stimuli at trial 150 (red dashed line in **(B)**). **(C)** Expected stay probabilities for pure model-free and pure model-based control. Only model-based control predicts the use of the transition structure of the task (common vs. rare trials) to make choices. Imagine in the example trial 2A that a subject chooses the horizontal ellipse in the first stage, then ends in the rare yellow second stage and selects the “flash-like” stimulus, which is then rewarded. Model-free control predicts a repetition of the first stage choice (horizontal ellipse) in the next trial. However, model-based control predicts a switch to the opposing first stage stimulus (vertical ellipse) in the next trial, because this stimulus has a higher probability in leading to the yellow second stage, where the subject can again choose the flash-like stimulus. Thus, model-free and model-based control make distinct predictions about next trials’ first stage choice behavior after rare trials.

We used a two-step Markov decision task as previously described (Daw et al., 2011; Voon et al., 2015, 2017), see Figure 1. The logic of the task is, that one can infer the extent to which a subject uses knowledge of the task structure to infer environmental contingencies, which formalizes model-based planning.

The task consists altogether of 201 trials, each trial consisting of two stages where subjects have to choose between two stimuli. Each choice at the first stage is associated with a certain transition frequency to the stimulus pair at the second stage.

For instance, whereas the choice of the horizontal ellipse commonly (in 70% of choices) leads to the green second stage stimulus pair but rarely to the yellow stimulus pair (30% of choices), the vertical ellipse commonly (in 70% of choices) leads to the yellow stimulus pair but rarely (in 30% of choices) to the green stimulus pair (Figure 1A). Selection of the second stage stimulus leads to monetary outcome (0.20 EUR, Figure 1B) according to slowly changing probabilities and subjects are instructed to maximize their reward by choosing stimuli that at a given moment are associated with reward.

The transition frequency from first to second stage reflects environmental contingencies (task structure) and the extent to which subjects incorporate this knowledge in their choices determines their “model-based behavioral signature” (Figure 1C). For example, if a first-stage choice is followed by a rare transition and individuals ultimately receive a reward at the second stage, they should be less likely to repeat that choice on the next trial because the alternative choice has a higher probability of returning the subject to that valuable second-stage state.

### Prejudice Questionnaire

Subjects completed the BSPS, a questionnaire including 20 items on prejudices toward Turkish migrants. Previous studies had identified two independent factors behind the items (Pettigrew and Meertens, 1995). One factor was related to blatant prejudices, which is considered the “traditional” type of prejudice. The other factor was related to subtle prejudices, which are considered to be a more “modern” form. Example items for both scales are outlined in Table 2.

**TABLE 2:** Example items for blatant and subtle prejudices of the blatant and prejudice scale (Pettigrew and Meertens, 1995).

	Example item	Factor	Number of items
	***Blatant prejudices***		
1.	Turkish migrants have jobs that the Germans should have	Threat and rejection	6
2.	I would be willing to have sexual relationships with a Turkish migrant	Intimacy	4
	***Subtle prejudices***		
3.	Turkish migrants who live here teach their children values and skills that are different from those that are necessary for a successful life in Germany	Traditional values	4
4.	How similar or different are Turkish migrants living here compared to other Germans in terms of the values that they convey to their children?	Cultural differences	4
5.	How often have you felt admiration for Turkish migrants living here?	Positive emotions	2

Both factors include 10 items each, relating to different categories. Blatant prejudice includes (1) threat/reject, and (2) intimacy items; subtle prejudice includes (1) traditional values, (2) cultural differences, and (3) positive emotion items. Participants’ answered using a 4-point Likert-type scale. Prior to summation, and in line with Pettigrew and Meertens (1995) item scores relating to “strongly disagree,” “somewhat disagree,” “somewhat agree,” and “strongly agree” were scaled as 1, 2, 4, and 5, respectively, with higher scores indicating greater prejudice. The final sum scores on the Blatant and Subtle scale ranged from 10 to 50. A higher sum score for each of the two scales relates to stronger prejudices.

### Analyses

We performed three sets of analyses that were all conducted to test how prejudices relate to the balance between model-free and model-based control. The first was a mixed effects logistic regression where first-stage choices (stay/switch) were regressed on the previous trial outcome (reward/no reward), transition (common/rare) and log scaled prejudice [(A) subtle and (B) blatant)]. Within-subject factors (intercept, main effect of outcome, main effect of transition and their interaction nested in subjects) were taken as random effects.

Secondly, we performed reaction time (RT) analyses. We regressed transition (common, rare) and log scaled prejudice [(A) subtle and (B) blatant)] on second stage RTs to test our hypothesis that model-based control was reduced in subjects with strong prejudice. The rationale for this analysis is that if subjects show reduced model-based control, they should also show less discrimination between common and rare trials in their second stage RTs. We (Sebold et al., 2016; Kroemer et al., 2019) and others (Deserno et al., 2015a) have shown that these RT effects are indeed associated with individual model-based control (which we also confirmed in this study, Supplementary Material 8). RTs faster than 250 ms were excluded from further data analysis to approach normal distribution and to exclude trials in which subjects performed implausibly fast (Brown et al., 2021).

**FIGURE 2:**
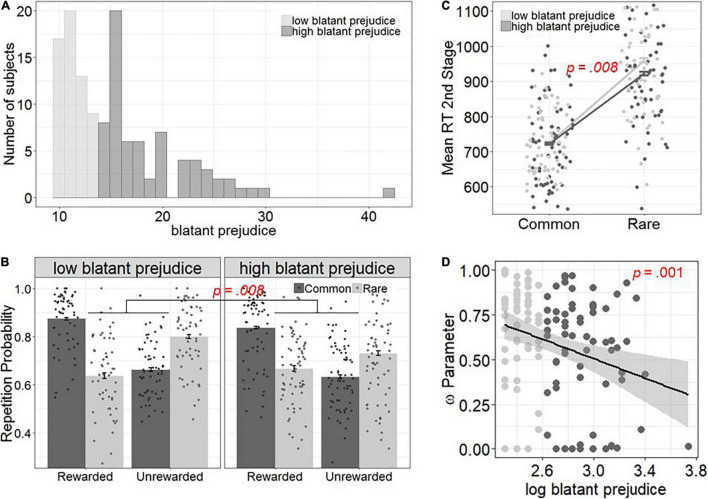
Association between blatant prejudices and different measures of the balance between model-free and model-based control. For visualization we performed a median split of the blatant prejudice scale to disentangle subjects with low (questionnaire sum score <14) and high (>=14) prejudices. **(A)** Distribution of the blatant prejudice scale prior to log scaling and according to the median split. **(B)** Observed choice behavior for subjects with high and low prejudices. Subjects with high prejudices showed a significant decrease in model-based behavior. **(C)** Second stage RT analyses as a function of blatant prejudice and transition. Subjects with high prejudices made faster responses in rare compared to common trials, indicating reduced model-based control. The regression lines in **(C,D)** depict the linear smoothed conditional means. **(D)** The negative association between blatant prejudice and model-based control was also supported by the computational analyses, as higher ω parameters were associated with lower blatant prejudice.

The third analysis was the fit of the original Daw et al. (2011) reinforcement learning model, a seven-parameter hybrid model, to the data (see Supplementary Material 1). Note, that prior to data analysis, we verified that this model was the best fitting model to our data (see Supplementary Material 2) and sufficiently captured the behavioral data (Supplementary Material 3). We used an expectation maximization algorithm to find maximum *a posteriori* estimates of the parameters. Practically, the model consists of two different sets of parameters: the reinforcement learning parameters that capture the internal learning and evaluation processes, and the response (softmax) parameters that transform the result of the internal valuations to choices. We hypothesized that prejudices would specifically influence learning parameters but had no hypothesis on how it could affect softmax parameters. The learning parameters in the hybrid model included two learning rate parameters (α1 and α2 for first and second stages, respectively), the weighting parameter ω (indicating the balance between model-free and model-based control with values closer to 0 indicating imbalance toward model-free and values closer to 1 imbalance toward model-based control), and the eligibility trace parameter λ from the model-free algorithm (indicating how strongly second stage outcomes update first stage action values with higher values indicating a stronger update). Based on our assumption that prejudices are associated with the balance between model-free and model-based control, we hypothesized that prejudice would be negatively associated with the weighting parameter ω. To this end, we regressed the weighting parameter ω (z-scaled) on the Prejudice Questionnaire data (log transformed to reduce impact of outliers, see Figure 2A). As associations with other parameters were also likely, we performed the same analyses with the remaining four reinforcement parameters (α1, α2, ω, λ). *P*-values smaller than 0.0125 (Bonferroni correction for four correlations) were considered significant.

As cognitive speed, working memory and impulsivity have been associated with the balance between model-free and model-based control (Otto et al., 2013, 2015; Schad et al., 2014; Deserno et al., 2015b; Gillan et al., 2016; Friedel et al., 2017), we added performance in the digit symbol substitution test (DSST) and Digit Span backward (DS) and scores in the Barratt Impulsiveness Scale [BIS-15; (Patton et al., 1995)] as index of cognitive speed, working memory and impulsivity, respectively, as covariates were not directly relevant to our research questions but still explains variance in behavior of the task.

Regression analyses were conducted using generalized linear mixed-effects models implemented with the lme4 package (Bates et al., 2015) in the R programming language, version 3.1.2^1^. For all regression analyses, we ensured that there was no multicollinearity (as defined VIF > 10) between the predictor variables. Furthermore we ensured, that there were no outliers (as defined by values >3 SD different from mean) in our linear predictor variables. For the logistic regression analysis we visually checked for the linear relationship between the prejudice variable and the logit of the stay/switch behavior. For the analyses of the RT data, we visually examined the residuals of the linear regression models for major deviations from linearity, normality and homogeneity.

As measures of effect size, we report coefficients of determination (semipartial *R*^2^β) using the procedure for linear mixed effects models implemented in the r2glmm package (Edwards et al., 2008; Jaeger et al., 2017). Computational modeling was performed in Matlab (2014) (version 8.3., 2014a).

## Results

### Stay-Switch Behavior

The results of the logistic regression without the prejudice term showed a main effect of outcome [*B* = 0.382, *p* < 0.0001, *R*^2^β = 0.003, 95% CI = (0.005, 0.002)], a main effect of transition [*B* = 0.344, *p* < 0.0001, *R*^2^β = 0.003, 95% CI = (0.004, 0.001)], and an interaction between outcome and transition [*B* = 2.101, *p* < 0.0001, *R*^2^β = 0.024, 95% CI = (0.028, 0.020)]. Hence, as reported previously (Daw et al., 2011; Sebold et al., 2019), subjects in this sample showed individual variation regarding their respective mixture between model-free and model-based learning (Figure 2B).

When adding blatant prejudices as between subject effect in the regression, blatant prejudices interacted with transition and outcome [3-way interaction; *B* = −0.08, *p* = 0.007, *R*^2^β = 0.001, 95% CI = (0.002, 0.000) Figure 2B], but not with transition (*p* = 0.078) nor outcome (*p* = 0.182) alone.

The results of the full regression analysis are outlined in Table 3. *Post hoc* regression analyses, in which we regressed prejudice onto the interaction between Reward and Transition (fixed plus random effect) suggested a negative association between model-based control and blatant prejudices [*B* = −0.23, *p* = 0.005, *R*^2^β = 0.001, 95% CI = (0.002, 0.001)]. Thus, subjects reporting higher blatant prejudice also showed reduced model-based control. In contrast, subtle prejudice did not interact significantly with outcome (*B* = 0.002, *p* = 0.718), transition (*B* = −0.000, *p* = 0.971), or the outcome and transition interaction (*B* = −0.020, *p* = 0.345).

**TABLE 3:** Result of the full regression analysis, indicating that blatant prejudice covaries with model-based control.

	Estimate	SE	*z*-value	*p*-value
Intercept	1.619	0.468	3.457	**0.001**
Transition	0.558	0.130	4.278	**0.000**
Outcome	0.191	0.155	1.238	0.216
Blatant prejudice	–0.018	0.012	–1.560	0.119
Cognitive speed	–0.003	0.020	–0.131	0.896
Working memory	–0.007	0.010	–0.712	0.477
Impulsivity	0.009	0.020	0.485	0.628
Transition × outcome	3.334	0.491	6.796	**0.000**
Transition × blatant prejudice	–0.014	0.008	–1.765	0.078
Outcome × blatant prejudice	0.013	0.009	1.333	0.182
Transition × outcome × blatant prejudice	–0.080	0.030	–2.683	**0.007**

*p-values < 0.05 are displayed in bold.*

### Reaction Time

We next regressed subtle and blatant prejudices and transition on second stage RTs. This revealed a significant main effect of transition [*B* = −314.58, *p* = < 0.0001, *R*^2^β = 0.028, 95% CI = (0.032, 0.024)]. Thus subjects slowed down after rare trials. Beyond this, we found an interaction between blatant prejudice and transition [*B* = 5.976, *p* = 0.008, *R*^2^β = 0.003, 95% CI = (0.004, 0.002), Figure 2D]. Thus, subjects with low prejudice slowed down more after a rare transition compared to a common transition, which is suggestive of more model-based inference (Deserno et al., 2015a; Sebold et al., 2016). We found no significant interaction between subtle prejudice and transition on such second stage RTs (*p* = 0.338).

### Computational Modeling

The correlation between subtle and blatant prejudice and the four reinforcement parameters mirrored our results from the regression analysis. There was a significant negative association blatant prejudice and ω [*B* = −0.852, *p* = 0.003, *R*^2^β = 0.068, 95% CI = (0.173, 0.009), Figure 2C], indicating that a relative shift from model-based toward model-free control was associated with higher levels of blatant prejudice. Blatant prejudice levels were not significantly associated with other reinforcement parameters from the computational model (Supplementary Material 4). No association between subtle prejudice scores and ω or other model parameters were found (all *p* > 0.05).

Crucially, all associations between model-based control and blatant prejudices remained significant after removal of the person with the most extreme blatant prejudice values (Behavioral data: *p* = 0.021; RT data: *p* = 0.025; Computational modeling: *p* = 0.007).

## Discussion

Prejudices and discrimination toward ethnic and cultural minorities can challenge social cohesion and contribute to social isolation and stress (Bhui, 2002; Heinz et al., 2019). Within a cohort of young male subjects, we here report that subjects with stronger prejudices toward Turkish migrants (the largest minority in Germany) also show reduced deliberative goal-directed, model-based decision-making. We demonstrate these associations by using different methodological approaches including choice (stay-switch behavior) and reaction time analyses as well as computational modeling. Crucially, these results could not be explained by interindividual differences in cognitive speed and impulsivity – measures that have repeatedly been associated with the balance between model-free and model-based control.

As prejudices and model-based control as computational mechanisms of decision-making have so far been investigated independently and by different researchers using different methodological approaches, this study is the first to link these domains. Our study suggests that individual differences in one domain predict individual differences in the other domain. Thus, this study adds to a recent proposal made by several researchers to incorporate computational methods in social psychology (Reiter et al., 2017; Tognoli et al., 2018; Yu et al., 2019).

According to a prominent theory, prejudices involve two facets: blatant and subtle prejudices (Pettigrew and Meertens, 1995). Blatant prejudices are also described as “hot” and “direct.” Although this definition cannot be falsified and might thus solely describe a phenomenological experience, it stresses their affective, rather impulsive component. Subtle prejudices, on the other hand, are described as “cool” and “indirect” and may represent a more rational component.

Some studies suggest that subtle prejudices have replaced blatant prejudices over the last decades (Pearson et al., 2009). In Germany, blatant prejudices have been a prominent problem since the early 90s, when some German cities such as Hoyerswerda, Solingen, and Rostock gained sad notoriety due to xenophobic violence (Lüdemann and Erzberger, 1994). Moreover, in recent years, the rise of the political far right has tended to make open racial statements toward migrants and other ethical minorities more common and publicly acceptable (Strauß, 2017; Sadeghi, 2019) across Germany.

Interestingly, in our study only blatant but not subtle prejudices were inversely associated with model-based control across all analyses. As model-based control is considered the more rational action selection strategy, this association suggests that subjects with a tendency to support affectively laden negative judgments also show a tendency to act less deliberative in a decision-making task. This finding has implications for interventions that targeted at changing prejudices across societies.

Several campaigns have aimed to reduce blatant prejudices. Targeting the morality of blatant prejudices has been shown to be particularly effective. For instance, inducing people to feel empathy by encouraging them to imagine what it might be like to be an outgroup person in a prejudiced society reduced prejudiced attitudes (Batson et al., 1997; Batson and Powell, 2003). In a previous study, we have also observed a negative correlation between empathy and blatant as well as subtle prejudices (Önal et al., 2021). Thus, empathy is one facet of morality that can be targeted to reduce prejudices. Applying a computational perspective, several recent studies have investigated the computational mechanism of empathy. For instance, learning to obtain rewards for others (as opposed to oneself) was shown to be accompanied by a unique neural reward prediction error (PE) signal in the subgenual anterior cingulate cortex (sgACC; Lockwood et al., 2015). Interestingly, subjects with higher trait empathy learned faster to obtain rewards for others and also showed stronger neural PE signals in the sgACC (Lockwood et al., 2016). In our study, however, trait empathy was not associated with model-based control, although it was negatively associated with blatant prejudices (Supplementary Material 7). Future studies should test, whether targeted interventions aiming at key learning mechanisms might potentially reduce stigmas and prejudices or increase empathy.

While the computational studies described above (Lockwood et al., 2015, 2016) focused on how actions maximize wins for others, empathy and moral behavior more generally also includes actions to minimize costs (e.g., harm) for others. A number of studies in the context of moral decision-making have focused on the computational basis of how individuals value harm to others. By using a computational model that quantifies the relative value subjects ascribe to pain for themselves vs. pain for others, it was shown, that most subjects show “hyperaltruistic” behavior in the way that they valued others’ pain more than their own pain (Crockett et al., 2014). This “hyperaltruistic” behavior was associated with slower responding when making decisions for others vs. oneself, indicating that moral decision-making is associated with the engagement of deliberative processes (Crockett et al., 2014). This is in line with our finding that less altruistic or moral behavior (as indicated by more prejudiced attitudes) is associated with a lack of deliberative processes (e.g., model-based control).

Future studies could investigate whether training model-based control decreases prejudices. While there is some recent evidence that model-based decision-making is rather insensitive to training (Grosskurth et al., 2019), one study demonstrated that training of the two-step task made individuals less resistant to distraction (Economides et al., 2015). However, in this latter study, trained subjects showed no shifts toward model-free control when their cognitive capacity was limited. On the other hand, monetary incentives were shown to boost model-based control in subjects with several psychiatric disorders (Patzelt et al., 2019). Thus, training of the two-step task might render individuals less prone to shifts toward model-free decision-making, and particular variants of task conditions may even shift individuals toward more model-based control. However, the effect of such interventions may be limited due to the observation, that subtle prejudices are more resistant to change compared to blatant prejudices (Herrero Olaizola et al., 2014).

Our study bears a number of limitations. First of all, we did not experimentally test how alterations in model-based control affect prejudices. Instead, our study is purely correlational and therefore limits any conclusion about causality. One previous study (Kurdi et al., 2019) demonstrated that explicit evaluations were sensitive to revaluations (as an index of model-based control), whereas implicit evaluations were not. This latter study used the same stimuli in the revaluation task and in the explicit/implicit measures (by means of ratings/IAT) and thus enabled a direct test how revaluation would alter implicit and explicit measures. One other study (Hackel et al., 2019) showed that social interactions were directly associated with model-free and model-based learning. In this study, subjects performed a variant of the two-step task, in which they had to learn the association between different financial advisors and high- or low-paying stocks and perform ratings of the different financial advisors after the task. Inspired by these experimental designs, future studies could directly test whether model-based control is associated with prejudice by using a social two-step task that involves interaction with individuals from an in and out -group by performing scores on implicit and explicit measures (e.g., IAT/ratings) of attitudes toward the in and out -group individuals after the task.

Second, we used the original version of the two-step task (Daw et al., 2011). Previous studies showed no correspondence between model-free reinforcement learning in this task and measures of habitual behavior in outcome devaluation tasks (Friedel et al., 2014; Gillan et al., 2015; Sjoerds et al., 2016). However, our results indicate an association between prejudices and model-based reinforcement learning, which did show the assumed correlation with goal-directed control in previous studies. Nevertheless, these previous findings support the recent notion to look beyond dual systems and dichotomies in behavioral control (Daw, 2018; Melnikoff and Bargh, 2018; Collins and Cockburn, 2020) calling for more sophisticated experimental approaches to be used in future studies, which allow a more fine-grained examination of behavior on the continuum from fully automatic to fully deliberate.

Another limitation of our study is that prejudices and model-based control were assessed on different days, thus raising the question of how state-dependent these measures are. However, as a previous study has indicated high test-retest-reliability for parameters from the two-Step task with short test-retest intervals of approximately 1 week (Brown et al., 2021) we believe that the assessment schedule might be negligible for the interpretation of the results. One further limitation of our study is, that our study sample consisted of an age and gender homogenous group, including only 21-year-old male subjects living in two major German cities. This potentially limits the generalizability of our study to cohorts with distinct demographic attributes. However, prejudices promoted by right wing political parties are particularly popular among male individuals (Goerres et al., 2018), and engagement in violent criminal acts against outgroup members is most likely to be observed during early adulthood (Kimmel, 2018; Carlsson et al., 2020), which stresses the importance of elucidating the cognitive mechanisms that underly prejudices in male young subjects. Interestingly, our study was recruiting across two German cities which substantially vary with regard to size and proportion of immigrants (Berlin predominates Dresden). Follow-up analyses (Supplementary Material 5) revealed that the negative associations between blatant prejudices and model-based control were not modulated by center effects. Thus, we assume that the association reported here is generalizable to urban environments with different demographic attributes and cultural diversities. Moreover, our results remained stable after we removed subjects who reported an immigrant background (Supplementary Material 6), suggesting that again our results can be generalized.

Our sample did not show a strong variance in the subscore of blatant prejudices (Figures 2A,B), and the distribution of this score was highly skewed, with many subjects showing very weak prejudices. Future studies in samples with higher levels of prejudice are warranted. Based on our sample size, we were limited to pure correlational analyses and could not apply structural equation models, which enable test of the precise direction.

In summary, we observed a significant association between reduced deliberative model-based decision-making and stronger blatant prejudice among young men in Germany. This was reflected in behavioral analyses and computational modeling of decision-making, in line with the hypothesis that prejudices can be habitually or “automatically” activated (Devine, 1989, 2001).

## Data Availability Statement

The raw data supporting the conclusions of this article will be made available by the authors, without undue reservation.

## Ethics Statement

The studies involving human participants were reviewed and approved by the Ethikkommission Charité – Universitätsmedizin Berlin. The patients/participants provided their written informed consent to participate in this study.

## Author Contributions

MS, AH, MG, and HC initiated the idea of the manuscript. HC, SN, SK-P, NM, AÖ, and MG gathered the data. HW, QH, FS, MR, MNS, and AH planned the study. MS, QH, and MG programmed the task. MS, HC, SK-P, and AH drafted the manuscript. MS performed all the analyses. All authors gave critical input to the analyses and final version of the manuscript.

## Conflict of Interest

The authors declare that the research was conducted in the absence of any commercial or financial relationships that could be construed as a potential conflict of interest.

## Publisher’s Note

All claims expressed in this article are solely those of the authors and do not necessarily represent those of their affiliated organizations, or those of the publisher, the editors and the reviewers. Any product that may be evaluated in this article, or claim that may be made by its manufacturer, is not guaranteed or endorsed by the publisher.

## Footnotes

1
cran.us.r-project.org
