# Supplementary material for: Stronger Prejudices Are Associated With Decreased Model-Based Control
Source: Front Psychol. 2022 Jan 5;12:767022. doi: 10.3389/fpsyg.2021.767022 (PMC8767058; doi:10.3389/fpsyg.2021.767022)
Supplement: Supplementary file 1 [file Data_Sheet_1.docx]

Supplementary Information:

Stronger Prejudices are associated with decreased model-based control

Sebold, M.^1,2^_,_ Chen H.^3^, Önal A.^1^, Kuitunen-Paul S.^4,5^, Mojtahedzadeh, N.^3^_,_Garbusow, M.^1^, Nebe, S.^6^, Wittchen, H-U.^7^, Huys Q.J.^8,9^, Schlagenhauf, F.^1,10^, Rapp, M.A.^2^, Smolka M.N.^3^, Heinz, A.^1^

^1^Department of Psychiatry and Psychotherapy, Charité – Universitätsmedizin Berlin, corporate member of Freie Universität Berlin, Humboldt-Universität zu Berlin, and Berlin Institute of Health, 10117 Berlin, Germany

^2^Department for Social and Preventive Medicine, University of Potsdam, 14469 Potsdam, Germany

^3^ Department of Psychiatry and Neuroimaging Center, Technische Universität Dresden, 01307 Dresden, Germany

^4^ Institute of Clinical Psychology and Psychotherapy, Technische Universität Dresden, 01187 Dresden, Germany

^5^ Department of Child and Adolescent Psychiatry, Faculty of Medicine, Technische Universität Dresden, 01307 Dresden, Germany

^6^ Zurich Center for Neuroeconomics, Department of Economics, University of Zurich, 8006 Zurich, Switzerland

^7^Ludwig Maximilians Universität, Department of Psychiatry & Psychotherapy, 80539 Munich, Germany

^8^ Division of Psychiatry, University College London, W1T 7NF London, United Kingdom

^9^ Max Planck UCL Centre for Computational Psychiatry and Ageing Research, University College London, WC1B 5EH London, United Kingdom

^10^Max Planck Institute for Human Cognitive and Brain Sciences, 04303 Leipzig, Germany

Corresponding Author:

Dr. Miriam Sebold, Charité Universitätsmedizin Berlin, Department of Psychiatry and Psychotherapy, Charitéplatz 1, 10117 Berlin, Germany

Phone: +49 30 450 517257; Mail: miriam.sebold@charite.de

# SI 1. Computational model

The algorithm of the 7-parameter hybrid model includes both model-based and model-free components, which allows for mapping state-action pairs to expected future values.

The model-free strategy is computed using the SARSA (λ) temporal difference learning. At each stage i of each trial t, the value for each state-action pair was calculated as follows:

*Q_TD_ (s_i,t_,a_i,t_) = Q_TD_ (s_i,t_,a_i,t_) + α_i_ δ_i,t_*

where δi,t = ri,t + QTD (si+1,t , ai + 1,t) - QTD (si,t ,ai,t) and αi is a free learning parameter. Different learning rates α1 and α2 for the two task stages were estimated. The reinforcement eligibility parameter (λ) determines the update of the first-stage action by the second-stage prediction error as follows:

QTD (s1,t , ai1,t) = QTD (s1,t ,a1,t) + a1λδ2,t .

The model-based reinforcement-learning algorithm was computed by mapping state-action pairs to a transition function and assuming that participants choose between two possibilities, as follows:

P(SB ⏐SA, aA) = 0.7, P(SC ⏐SA, aB) = 0.7 for common

and

P(SB ⏐SA, aA) = 0.3 P(SC ⏐SA, aB) = 0.3 for rare transitions,

where S is the state (first stage: SA; second stage: SB and SC), and a is the action (two actions: aA and aB) at a given state. The action value (QMB) was computed at each trial from the estimates of the transition probabilities and outcomes and was defined for the first stage as follows:

*Q_MB_ (s_A_,a_i_) = P(s_B_|s_A_,a_i,_) max_a_ Q_TD_(s_B_,a) + P(s_C_|s_A_,a_i,_) max_a_ Q_TD_(s_C_,a)*

Finally, to connect values to choices, the weighted sum of the model-free and model-based values was computed for the first stage as defined:

*Q_net_(s_A_,a_j_) = w Q_MB_(s_A_,a_j_) + (1-w) Q_TD_(s_A_,a_j_)*

where w is the weighting parameter. Assuming that two approaches coincide at the second stage, and that QMB = QTD, at the second stage Qnet= QMB = QTD. Then, the probability of a choice is the softmax equation for

Qnet:

*Σ_a’_ exp (β_i_[Q_net_(s_i,t_,a) + p * rep(a)])*

*P(a_i,t_ = a|s_i,t_) = exp (β_i_[Q_net_(s_i,t_,a) + p * rep(a)]) /*

where the free inverse temperature parameters (βi) control the choice randomness, and p captures perseveration (p > 0) or switching (p < 0) in the first-stage choices. In total, the hybrid model contains 7 free parameters (β1, β2, α1, α2, λ, p, ω), with special

cases of pure model-based (ω = 1) and model-free (ω = 0) models.

# SI 2. Model comparison

Following previous study (Sebold et al., 2017), we fitted two alternative models to our choice data: 1) a model-free algorithm SARSA (λ), which only captures a main effect of outcome on first stage choices, and 2) a pure model-based algorithm, which considers the interaction between outcome and transition frequencies, but does not capture a main effect of outcome on first stage choices. The overarching aim of these alternative model fittings was the subsequent model comparison, where we aimed to identify the best fitting algorithm across subjects. Therefore, we subjected individual model evidences (integrated log-likelihoods) for all three models to a Bayesian model selection procedure. Indeed, the hybrid model was the best fitting model with lowest BIC scores (Hybrid:53513; Model-free:55426; Model-based:54085) and highest Exceedance Probability (Hybrid:0.8024 ; Model-free:0.0561; Model-based:0.1416), SI Figure 1.


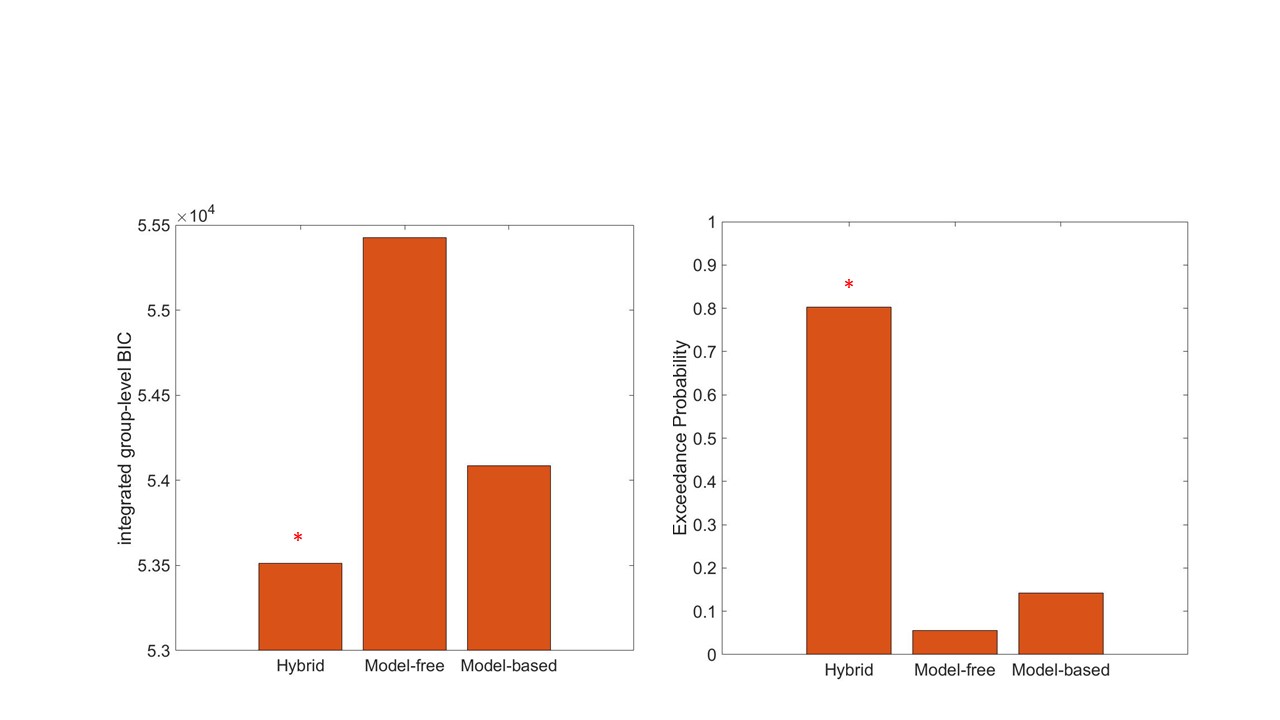


SI Figure 1: Results of the Bayesian model comparison procedure. The hybrid model was the best-fitting model to the data.

# SI 3. Simulation data

To ensure that the chosen model indeed captured the observed data, we additionally generated simulated first and second choice data for each subject based on the inferred computational parameters. For each subject, we computed 100 simulation data sets. Consequently we compared mean model-free (main effect of outcome) and model-based (interaction between outcome and transition) scores for all subjects between real data and simulated data. This comparison indicated high correlation coefficients for both model-free (r=.81, *p*<.0001) and model-based (r=.85, *p*<.0001) scores, indicating that the computational model indeed captures the behavioral data across subjects (SI Figure 2).


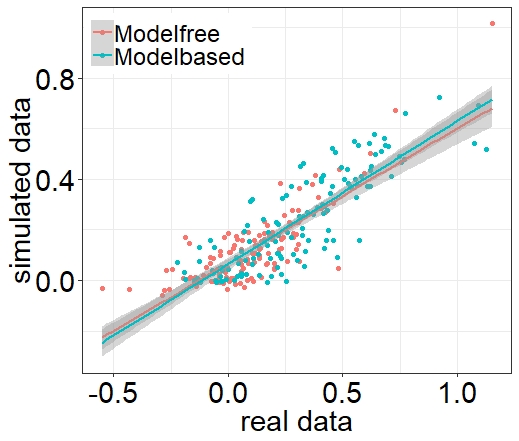


SI Figure 2: Association between real data and simulated data from the inferred computational parameters.

# SI 4. Blatant prejudice and other comp. Model parameters

Although our hypothesis focused on the balance between model-free and model-based control and therefore on the computational parameter ω, for exploratory purposes we also tested associations with all other model parameters (SI Figure 1). However, none of the remaining reinforcement parameters (α1 (*p* = .284), α2 (*p* = 931), λ (*p* = .705)) or softmax parameters (β1 (*p* = .308), β2 (*p* = .057), r (*p* = .294)) were associated with blatant prejudice (SI Figure 3). Also, In line with the logistic regression results, ω was not associated with subtle prejudice (*p* = 0.728), and neither were other model parameters (α1 (*p* = .465), α2 (*p* = .645), λ (*p* = .728), β1 (*p* = .428), β2 (*p* = .204), r (*p* = .152)).


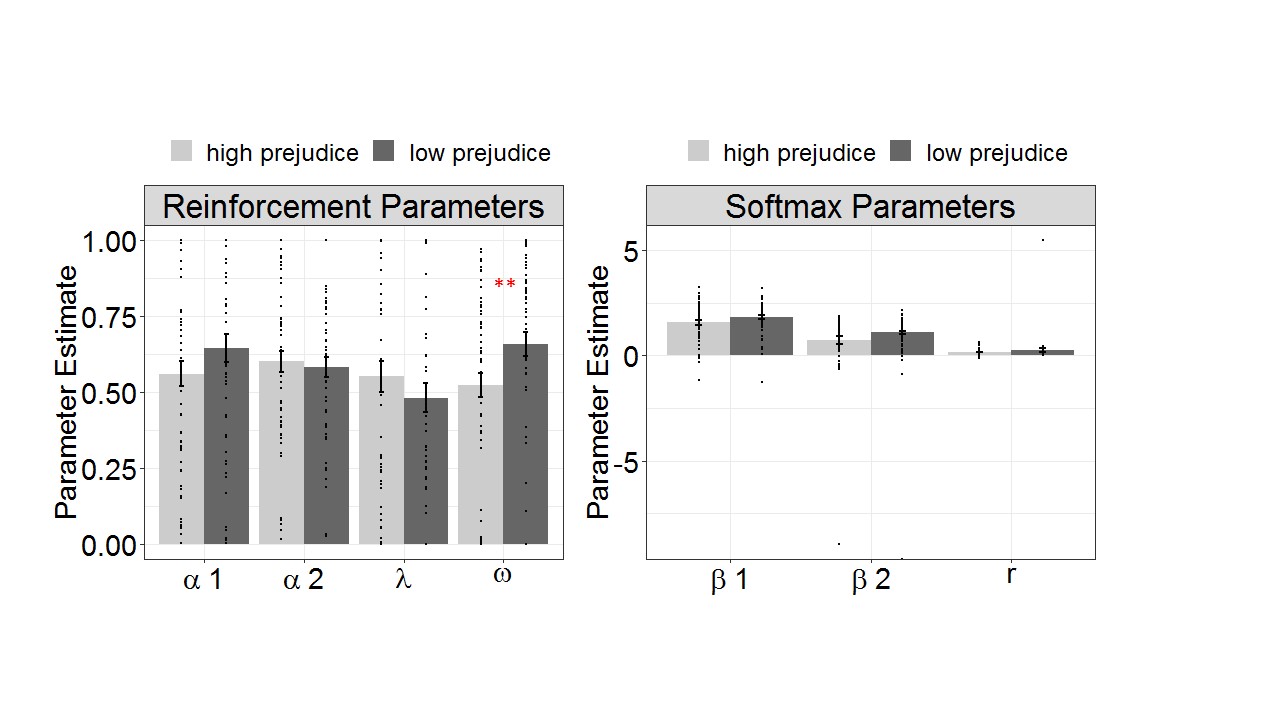


SI Figure 3: Association between blatant prejudice and the remaining reinforcement parameters (left) and the softmax parameters (right). Beta values were log transformed due to extreme outliers.

# SI 5. Center effects

Our cohort consisted of participants from 2 German cities (Berlin: n = 60; Dresden: n = 67). During study completion, Dresden experienced a particular rise of the far right party^1,2^. Thus, we performed follow-up analyses to test whether the here reported association between blatant prejudice and model-based control were particularly driven by subjects from Dresden. To this end we added site as an additional factor in our linear model to predict the computational parameter ω from blatant prejudices and tested for interaction or main effects. However, this analysis revealed no significant main effect of site (β = .011, p = .947) nor a significant interaction between site and blatant prejudice (β = .09, p = .387). Thus, ω was negatively associated with blatant prejudices across both sites (SI Figure 4).


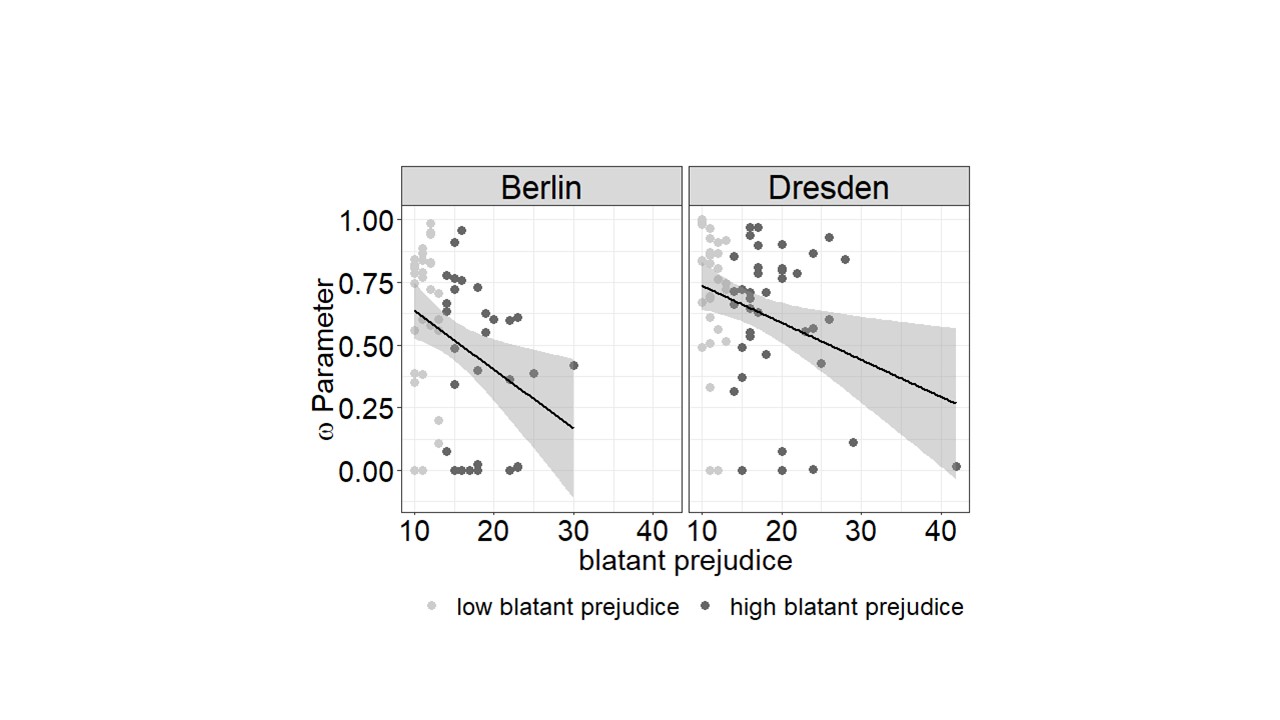


SI Figure 4: Site effects of the association between the computational parameter ω and blatant prejudice.

# SI 6. Effects of Migration background

The association between model-based control and prejudices remained stable after removing subjects with migration background from the sample (n=25). The regression analysis of the stay/switch behavior revealed a 3-way interaction between outcome, frequency and blatant prejudice (B = -.067, *p* = .0375). The regression analyses of 2^nd^ stage RTs as a function of transition and prejudices revealed a two was interaction (B = .006, *p* = .015). Moreover, the spearman correlation between the computational parameter ω and blatant prejudices revealed a significant association (ρ = -.25, *p* = .018). Thus, we conclude that the association between model-based control and blatant prejudices were not driven by those subjects reporting a Migration background.

# SI 7: Empathy, blatant prejudice and model-based control

As previous research by Lockwood, et al. ^3^ demonstrated, that trait empathy is associated with reinforcement learning signals, we further assessed trait empathy by means of the Saarbrücker Persönlichkeitsfragebogen^4^. To further investigate the association between prejudice, empathy and model-based control, we then tested whether trait empathy was associated with either blatant prejudice or model-based control (as indicated by the computational parameter ω). By performing these analyses, we found evidence for the former association (Spearman correlation between blatant prejudice and empathy score: ρ = -1.88, *p* = .034) but not for the latter (Spearman correlation between ω and empathy score: ρ = .105, *p* = .238).

# SI 8. 2nd stage RT effects and model-based control

To replicate previous findings, indicating an association between 2nd stage RT effects and model-based control ^5,6^, we performed an additional analysis in which we correlated 2nd stage RT effects (2nd stage RT in rare trials – 2nd stage RTs in common trials) with the computational parameter ω, indicating the balance between model-based and model-free control. This analysis revealed a strong positive association between theses indices (Pearson correlation: r = .5, p < .0001, SI Figure 5), indicating that 2nd stage RT effects indeed capture model-based signatures.


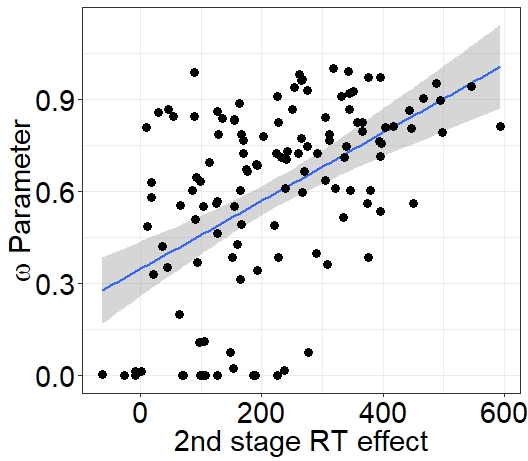


SI Figure 5: Association between 2^nd^ stage RT effects and the ω Parameter indicating the balance between model-free and model-based control

References

1 Reuband, K.-H. Wer demonstriert in Dresden für Pegida? Ergebnisse empirischer Studien, methodische Grundlagen und offene Fragen. *Mitteilungen des Instituts für Parteienrecht und Parteienforschung* **21**, 133-143 (2015).

2 Vorländer, H., Herold, M. & Schäller, S. *PEGIDA: Entwicklung, Zusammensetzung und Deutung einer Empörungsbewegung*. (Springer-Verlag, 2015).

3 Lockwood, P. L., Apps, M. A., Valton, V., Viding, E. & Roiser, J. P. Neurocomputational mechanisms of prosocial learning and links to empathy. *Proc Natl Acad Sci U S A* **113**, 9763-9768, doi:10.1073/pnas.1603198113 (2016).

4 Paulus, C. Der Saarbrücker Persönlichkeitsfragebogen SPF (IRI) zur Messung von Empathie: Psychometrische Evaluation der deutschen Version des Interpersonal Reactivity Index. (2009).

5 Sebold, M. *et al.* Don't Think, Just Feel the Music: Individuals with Strong Pavlovian-to-Instrumental Transfer Effects Rely Less on Model-based Reinforcement Learning. *J Cogn Neurosci* **28**, 985-995, doi:10.1162/jocn_a_00945 (2016).

6 Deserno, L. *et al.* Ventral striatal dopamine reflects behavioral and neural signatures of model-based control during sequential decision making. *Proc Natl Acad Sci U S A* **112**, 1595-1600, doi:10.1073/pnas.1417219112 (2015).
